# The Early Regenerative Events in the Human Tympanic Membrane After In Vivo Traumatization

**DOI:** 10.1002/lary.70717

**Published:** 2026-07-21

**Authors:** Elnaz Sepehri, Cecilia Engmér Berglin, Yan Liu, Per Olof Eriksson, Magnus von Unge, Julia Arebro

**Affiliations:** ^1^ Division of Ear, Nose and Throat Diseases, Department of Clinical Sciences, Intervention and Technology Karolinska Institutet Stockholm Sweden; ^2^ Department of Ear, Nose and Throat Diseases Karolinska University Hospital Stockholm Sweden; ^3^ Department of Otorhinolarygology Sophiahemmet Hospital Stockholm Sweden; ^4^ Department of Clinical Molecular Biology (EpiGen) Akershus University Hospital Lørenskog Norway; ^5^ Department of Surgical Sciences, Otorhinolaryngology Uppsala University Uppsala Sweden; ^6^ Institute of Clinical Medicine, Department of Otorhinolaryngology & Head and Neck Surgery University of Oslo Oslo Norway; ^7^ Centre for Clinical Research Västmanland, Uppsala University Västerås Sweden

**Keywords:** human, in vivo, regeneration, traumatization, tympanic membrane

## Abstract

**Objective:**

To observe the early regenerative response in the keratinocyte layer of the human tympanic membrane (TM) following in vivo traumatization.

**Methods:**

Thirteen human TMs were in vivo traumatized (with phenol or superficial scratching) and harvested after 20 h. Immunohistochemistry was performed using the specific epidermal progenitor cell markers CK19, p63, and α6‐integrin, and the keratinocyte proliferation marker Ki‐67. The staining appearance and intensity were evaluated and graded by two blinded evaluators for the different TM regions: the annulus, the malleus, the unattached pars tensa (UPT) close to the trauma (tUPT) and on the untraumatized (uUPT) side.

**Results:**

All protein markers were identified in all regions of the TM at 20 h post traumatization. CK19, p63, and Ki‐67 were upregulated in the regenerative regions at the annulus and malleus. Moreover, CK19 was significantly upregulated in the tUPT compared to the uUPT. α6‐integrin was, at the same timepoint, significantly upregulated in the tUPT in the TMs traumatized by scratching. Ki‐67, on the other hand, showed a tendency towards an upregulation in the uUPT.

**Conclusion:**

This study shows that traumatization activates a regenerative response in the human TM, activating key proliferative regions already at 20 h after traumatization. The results show an upregulated proliferation activity in the UPT in early healing and confirm the previously described regenerative regions of the annulus and malleus. These insights will be valuable for improving future TM healing strategies.

**Level of Evidence:**

N/A.

## Graphical abstract

At 20 h after in vivo traumatization a regenerative response in the human tympanic membrane is activated. The results show an upregulated proliferation activity in the UPT in early healing and confirm the previously described regenerative regions of the annulus and malleus.

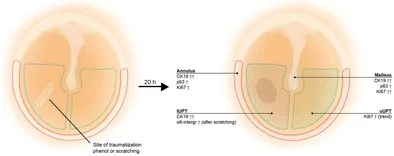

## 1 Introduction

The keratinized squamous epithelium of the tympanic membrane (TM) displays great capability regarding migration and proliferation of cells. This is evident since most tympanic membrane perforations (TMP) heal spontaneously within two to three weeks [1, 2]. In the absence of early spontaneous healing, a chronic TMP could evolve, resulting in long‐term discharge and hearing impairment. It is unknown why some TMPs do not heal spontaneously. In this aspect, understanding the early stages of the regeneration process in the human TM is of great importance for the development of methods that might prevent the formation or persistence of a chronic TMP.

Previous studies of acute TM healing are mainly histological or otomicroscopy observations in rodents. They report accumulating and migrating keratinocytes moving from the central portion of the TM laterally towards the perforation site, constituting part of the healing mechanism [3, 4, 5]. This is the same centrifugal direction as in the normal migration process of TM keratinocytes. This event also displays the importance of the outer epithelial layer being the first to close a TM perforation [3, 4, 5]. Though studying human chronic TMPs intra‐operatively, findings at the muco‐epithelial junction invaginated into the middle ear present an alternative theory of centripetal migration in the healing process [6]. In a study by von Unge et al., there were signs of keratin accumulation, to a much lesser extent, on the peripheral side of the TMP after induced trauma, advocating the possibility of a centripetal healing process [1, 2].

Various molecules have been suggested to be of importance for TM healing, such as plasminogen and fibroblast growth factor [7, 8, 9]. Internal factors are of importance to study in parallel with these external attempts. For instance, gene expression was shown to differ between skin and TM keratinocytes, with a more individualized migration capacity of the latter [10]. Moreover, the presence of progenitor and proliferator cells in the normal resting human TM by targeting specific epidermal protein markers was recently shown, using Ki‐67, p63, CK19, α6 and β1‐integrin [11].

The aim of the present study is to map the regenerative process in the human TM at 20 h after in vivo traumatization. Special attention is paid to comparison between expressions in four different regions of the pars tensa: at the annulus, the malleus and the unattached pars tensa (UPT), both at the side of the superficial trauma of the TM (tUPT) as well as on the untraumatized side (uUPT). Furthermore, attention is also paid to regenerative activity in three different anatomical levels: the inferior, intermediate and superior level.

## 2 Methods

### 2.1 Tympanic Membrane Samples

Healthy human TMs (*n* = 13), together with the attached malleus, were collected during lateral skull base surgeries involving blind sac closure of the external auditory canal, performed as part of a treatment for vestibular schwannoma (see experimental schedule, Figure 1). Each participant provided both verbal and written informed consent, in accordance with ethical approval granted by the Regional Ethical Review Board in Uppsala (Diary no. 2018/364). Twenty hours prior to harvest of the specimen during surgery, the TM was traumatized either by phenol application (*n* = 7) or by scratching the superficial surface of the TM (*n* = 6) with a sterile injection needle (0.7 mm outer diameter) until minor bleeding occurred, though not causing a perforation under a surgical microscope. The location of the trauma corresponded to the mid portion of the posterior half of the TM. The two different methods of traumatization were used to increase the generalizability of the generated findings. Immediately upon surgical harvest, the TMs, still with the malleus attached, were put in a standard 4% buffered formaldehyde solution for 2 days. Samples were subsequently put in 0.1 M ethylenediaminetetraacetic acid (EDTA) solution (37.2 g EDTA salt mixed with 1000 mL PBS) for decalcification of the malleus. The TMs were kept in the EDTA solution for 1 week, then dehydrated in serial dilution with ethanol according to standard histology procedure and embedded in paraffin. The TMs were oriented with their inferior part downwards and the head of malleus upwards to facilitate orientation when sectioning. The paraffin blocks with the TM samples were sectioned sequentially starting at the inferior side towards the umbo and continuing approximately 2000 μm superiorly to the level of umbo. The level of each TM section was controlled microscopically with hematoxylin and eosin staining to identify known structures of the TM. At every level of interest, multiple slides (up to 15) were cut and collected on Superfrost plus microscope slides (Thermo Fisher scientific, Waltham, MA USA) for subsequent immunohistochemistry (IHC) staining.

**FIGURE 1:**
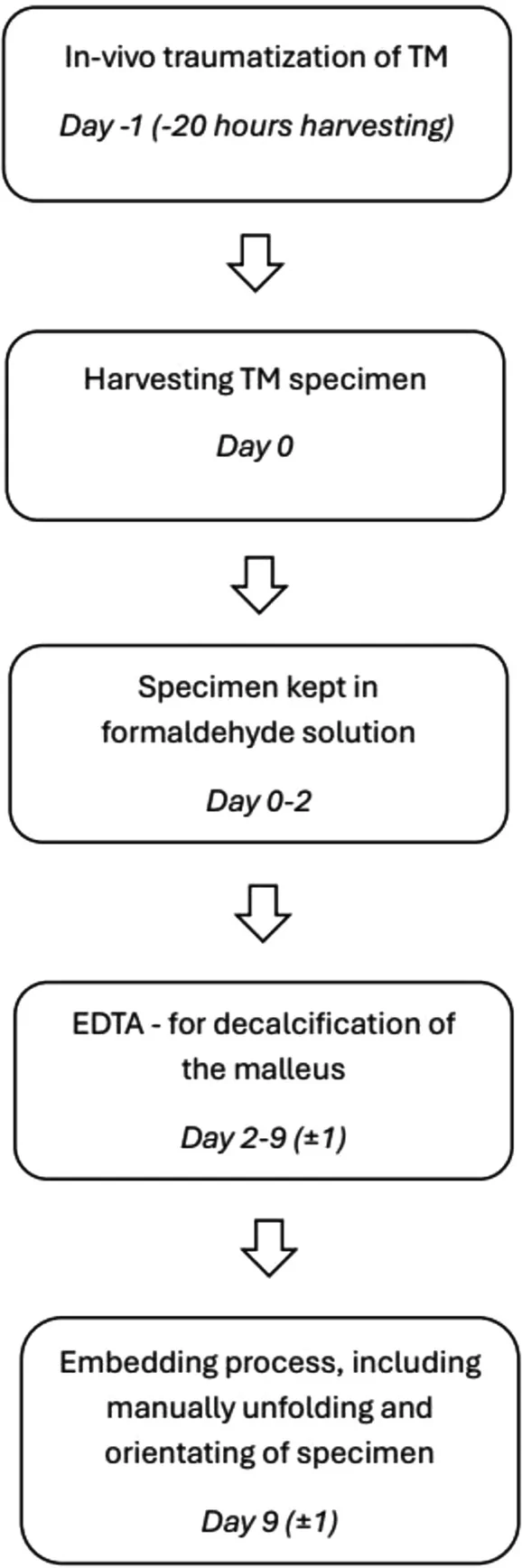
Experimental schedule.

### 2.2 Immunohistochemical Staining

The slides were heated for dissolving of the paraffin, preparing them for antibody staining with four different antibodies: anti‐Ki‐67, anti‐p63, anti‐CK19, and anti‐α6‐integrin. The product number of primary antibody, dilution and antigen retrieval solution pH used for IHC are summarized in Table S1. Four different regions of interest were defined: the pars tensa attached to the malleus (herein referred to as the malleus), the pars tensa at the annulus (herein referred to as the annulus), the pars tensa between the annulus and the malleus on the side of the trauma (herein referred to as the traumatized unattached pars tensa, tUPT), and the pars tensa between the annulus and the malleus on the untraumatized side (herein referred to as the untraumatized unattached pars tensa, uUPT) (Figure 2A). Three different anatomical levels were selected for investigation: the inferior, intermediate, and superior level (Figure 2B). The superior level was positioned at approximately 2000 μm superior to the umbo. The staining appearance and intensity of every antibody was graded and compared between the different levels within each region.

**FIGURE 2:**
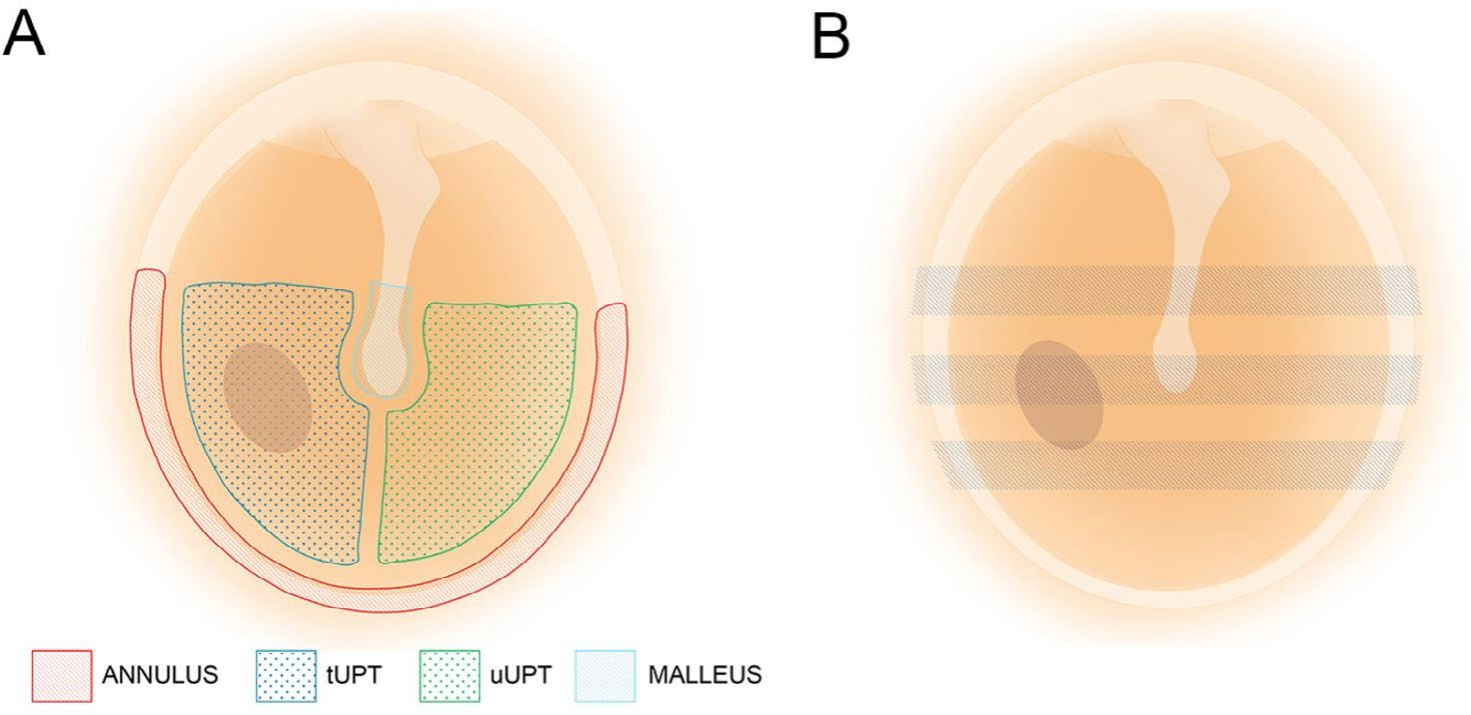
Schematic overview of the tympanic membrane. Filled brown circle indicate location of traumatization. (A) The four different regions evaluated: The annulus, malleus, the untraumatized unattached pars tensa (uUPT) and the traumatized UPT (tUPT). (B) The tympanic membrane divided into levels marked with horizontal gray shading: Inferior (inferior to the level of umbo), intermediate (at the same level as the umbo), and superior (superior to the level of the umbo) level. [Color figure can be viewed in the online issue, which is available at www.laryngoscope.com]

The staining protocol was formed by titrating for each antibody where a predetermined dilution was unavailable from the producer. Positive and negative controls were made for each antibody [12]. Dako autostainer Link 48 (Agilent Technologies Inc., Santa Clara, CA 95051 USA) was used with a standard program for staining. The slides were then scanned by an Aperio ScanScope AT scanner (Vista, CA 92081 USA). Aperio ImageScope was used as software for staining evaluation.

### 2.3 Evaluation of Antibody Staining

Evaluation was made single‐blinded by two external otologists, who were initially not involved in the study but had sufficient experience in TM histology. To begin with, multiple reference slides for each antibody were presented to the evaluators to help them recognize staining patterns and tympanic membrane morphology, enabling a consensus on the grading criteria. Each slide was then displayed for and subsequently scored by each evaluator independently and blinded to each other. A score was given for each defined region of each slide: the annulus, tUPT, uUPT, and malleus region (Figure 2A). The score range was from 0–3 depending on both the antibody staining intensity and/or frequency of positively stained cells. Scoring interpretation: 0 = no staining, 1 = low grade of staining, 2 = medium grade of staining, and 3 = frequent and/or intense grade of staining.

The results of the scoring were evaluated with weighted Cohen's kappa, yielding a coefficient (κ_w_) to measure inter‐rater reliability between the two raters to quantify the qualitative measures. Grading of antibody staining was made on an ordinal scale (0–3), motivating the usage of the weighted Cohen's kappa [13]. Group differences in antibody staining scores were statistically analyzed using one‐way ANOVA followed by Tukey's post hoc test for multiple comparisons. In cases where the evaluators assigned differing scores, the average of their assessments was used. A *p*‐value ≤ 0.05 was regarded as indicative of statistical significance (*p < 0.05, **p < 0.01, ***p < 0.001, ****p < 0.0001). Statistical analyses were performed using GraphPad Prism software (version 6.0, GraphPad Software, La Jolla, CA). All data are shown as mean ± standard error of mean. For figures with bars, *n* equals the number of slides.

## 3 Results

Of the 13 human TMs, seven were traumatized with phenol application and six by superficial needle scratching. In total, 141 slides had appropriate quality for further analysis regarding antibody staining. This included 116 slides for the annulus, 64 slides for the tUPT, 130 slides for the uUPT, and 93 slides for the malleus region. Among these slides, all 13 TMs were represented.

### 3.1 Inter‐Rater Reliability

Analysis of the weighted Cohen's kappa was performed for evaluation of the inter‐rater reliability regarding scoring of the slides stained with antibodies between the two evaluators, generating *κ*_w_ = 0.90 (Table 1). For the kappa calculations, including each region calculated separately, see Table S2. The grading difference between the two evaluators was never more than one step in range except for one single slide. The coefficient was considered almost perfect according to Landis et al., and the mean of the scoring results was then used for further statistical analyses [13].

**TABLE 1:** Analysis of inter‐rater reliability, evaluated with weighted Cohen's kappa coefficient.

Region	Value (*κ*_w_)
All regions	0.90
Annulus	0.90
Malleus	0.97
tUPT	0.74
uUPT	0.89

*Note:* Interpretation of *κ*_w_ results: 0.01–0.2 = poor, 0.21–0.4 = fair, 0.41–0.6 = moderate, 0.61–0.8 = substantial, 0.81–1.00 = almost perfect [13].

Abbreviations: κ_w =_ Cohen's kappa coefficient.

### 3.2 Presence of Proliferative and Stem Cell Markers in the Tympanic Membrane After Traumatization

The stem cell marker CK19 was identified in all four studied regions of interest: the annulus, the uUPT, the tUPT, and the malleus (Figure 3A–C). Twenty hours after the trauma, the staining scores in the annulus (*p* < 0.0001) and malleus region (*p* < 0.0001), but also in the tUPT (*p* < 0.0001) close to where the trauma had been performed, were significantly higher than that of the uUPT (Figure 3D). The staining score of CK19 in the UPT close to the trauma (tUPT) was comparable with the levels of CK19 in the annulus and in the malleus (Figure 3D).

**FIGURE 3:**
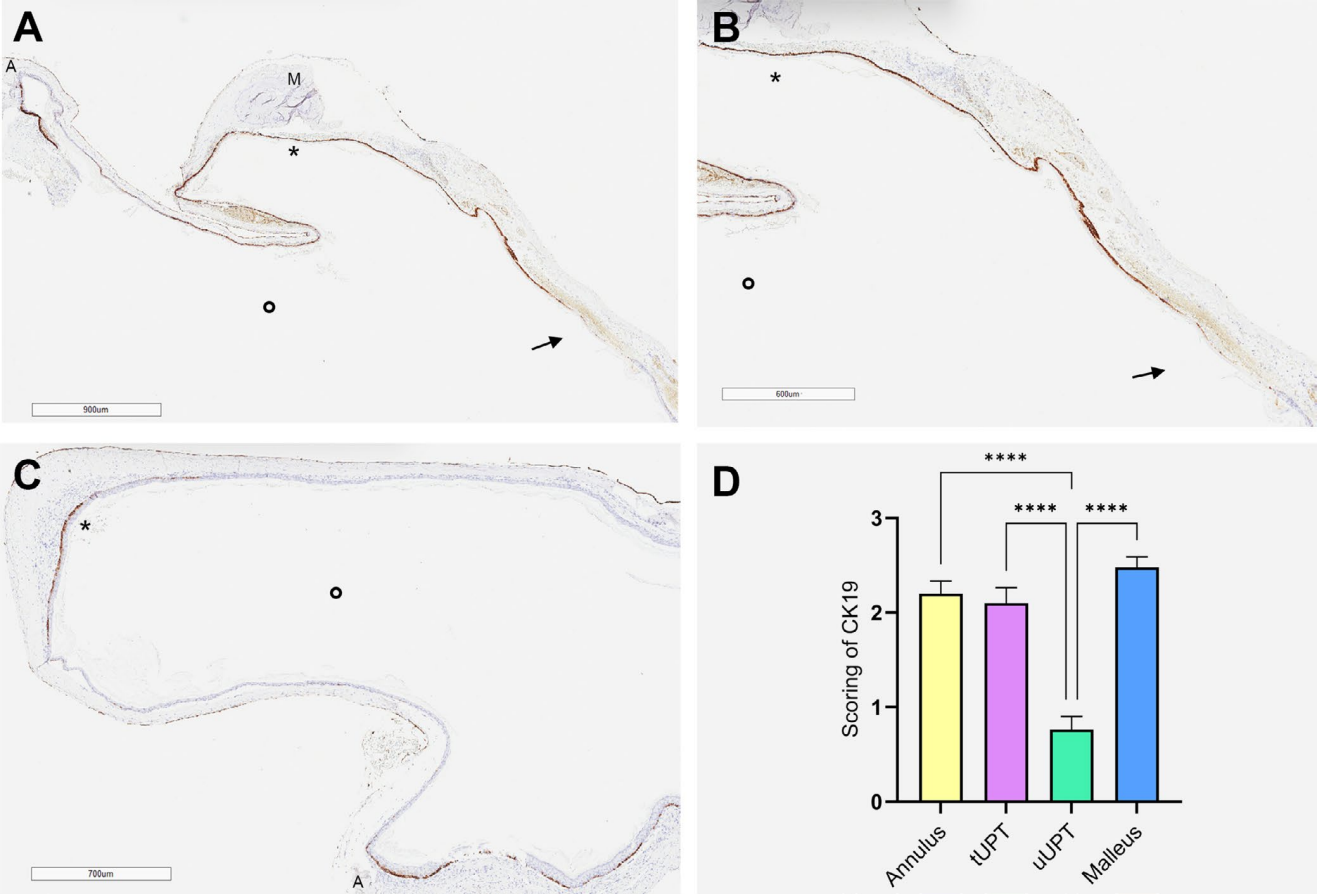
CK19 in the human tympanic membrane after traumatization. (A‐C) Immunohistochemical staining of CK19 in the tympanic membrane 20 h after traumatization. Open circle = external auditory canal, M = handle of malleus, A = annulus, star = region of malleus, arrow = site of traumatization. (D) Evaluation of CK19 staining of tympanic membranes. Each value within the bars (*n* = 15–34) represents one slide with mean scoring, with standard error of mean (*p < 0.05, ****p < 0.0001). UPT = unattached pars tensa, tUPT = traumatized UPT, uUPT = untraumatized UPT. [Color figure can be viewed in the online issue, which is available at www.laryngoscope.com]

The proliferative marker Ki‐67 was identified in all four regions studied: the annulus, the uUPT, the tUPT, and the malleus (Figure 4A–C). Twenty hours after the trauma, the staining scores were significantly higher in the annulus (*p* = 0.0035) and malleus (*p* < 0.0001) regions, compared to the tUPT (Figure 4D). The staining score was further significantly higher in the malleus (*p* = 0.0036) compared to the uUPT. However, there was no significant scoring of Ki‐67 in the annulus (*p* = 0.3945) when comparing with the uUPT.

**FIGURE 4:**
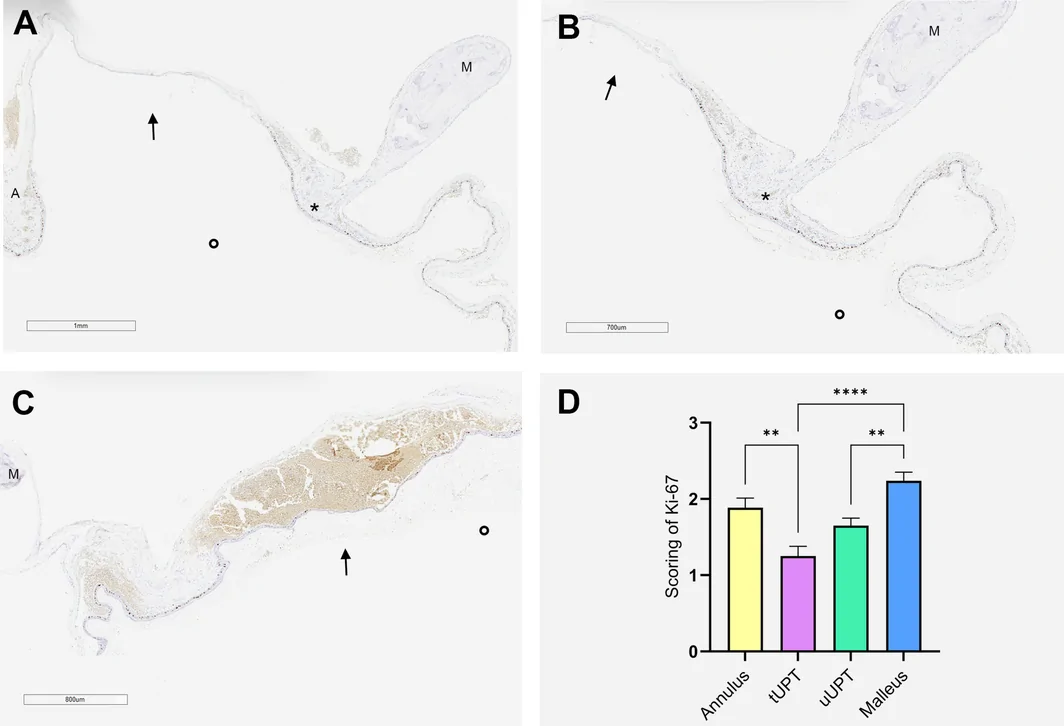
Ki‐67 in the human tympanic membrane after traumatization. (A‐C) Immunohistochemical staining of Ki‐67 in the tympanic membrane 20 h after traumatization. Open circle = external auditory canal, M = handle of malleus, A = annulus, star = region of malleus, arrow = site of traumatization. (D) Evaluation of Ki‐67 staining of tympanic membranes. Each value within the bars (*n* = 16–31) represents one slide with mean scoring, with standard error of mean (*p < 0.05, **p < 0.01, ****p < 0.0001). UPT = unattached pars tensa, tUPT = traumatized UPT, uUPT = untraumatized UPT. [Color figure can be viewed in the online issue, which is available at www.laryngoscope.com]

Upon 20 h of trauma, the stem cell marker p63 was identified in all four studied regions of interest (Figure 5A–C). The staining score was significantly higher in the annulus and malleus regions compared to the uUPT (*p* = 0.0002 and *p* = 0.0109 respectively) and tUPT (*p* = 0.0012 and *p* = 0.0248 respectively) (Figure 5D).

**FIGURE 5:**
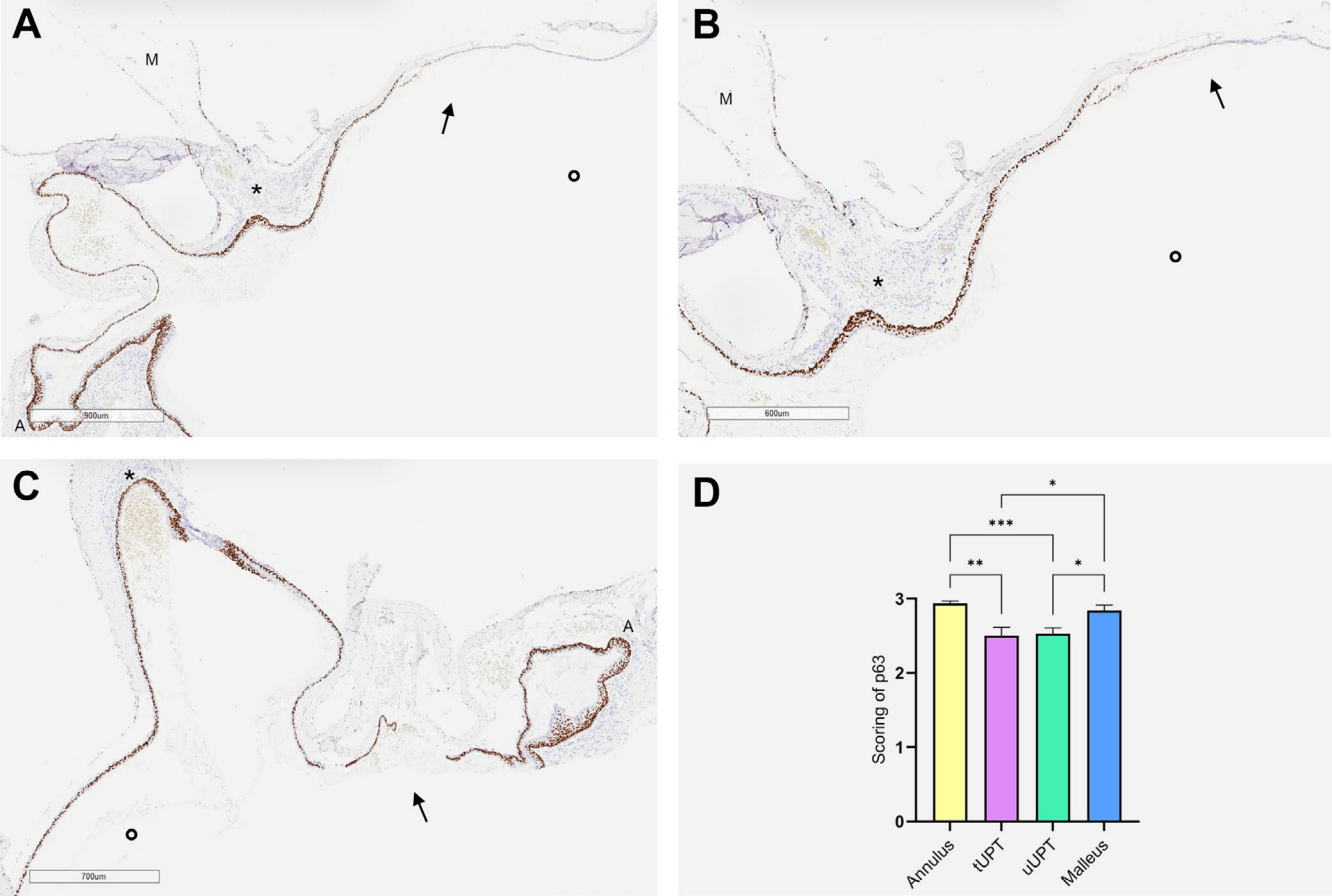
p63 in the human tympanic membrane after traumatization. (A‐C) Immunohistochemical staining of p63 in the tympanic membrane 20 h after traumatization. Open circle = external auditory canal, M = handle of malleus, A = annulus, star = region of malleus, arrow = site of traumatization. (D) Evaluation of p63 staining of tympanic membranes. Each value within the bars (*n =* 17–35) represents one slide with mean scoring, with standard error of mean (*p < 0.05, **p < 0.01, ***p < 0.001). UPT = unattached pars tensa, tUPT = traumatized UPT, uUPT = untraumatized UPT. [Color figure can be viewed in the online issue, which is available at www.laryngoscope.com]

Finally, integrin α6 could be identified in the annulus, the uUPT, the tUPT, and the malleus at 20 h after trauma (Figure 6A–C). There were no significant differences found in the scoring between the different regions (Figure 6D).

**FIGURE 6:**
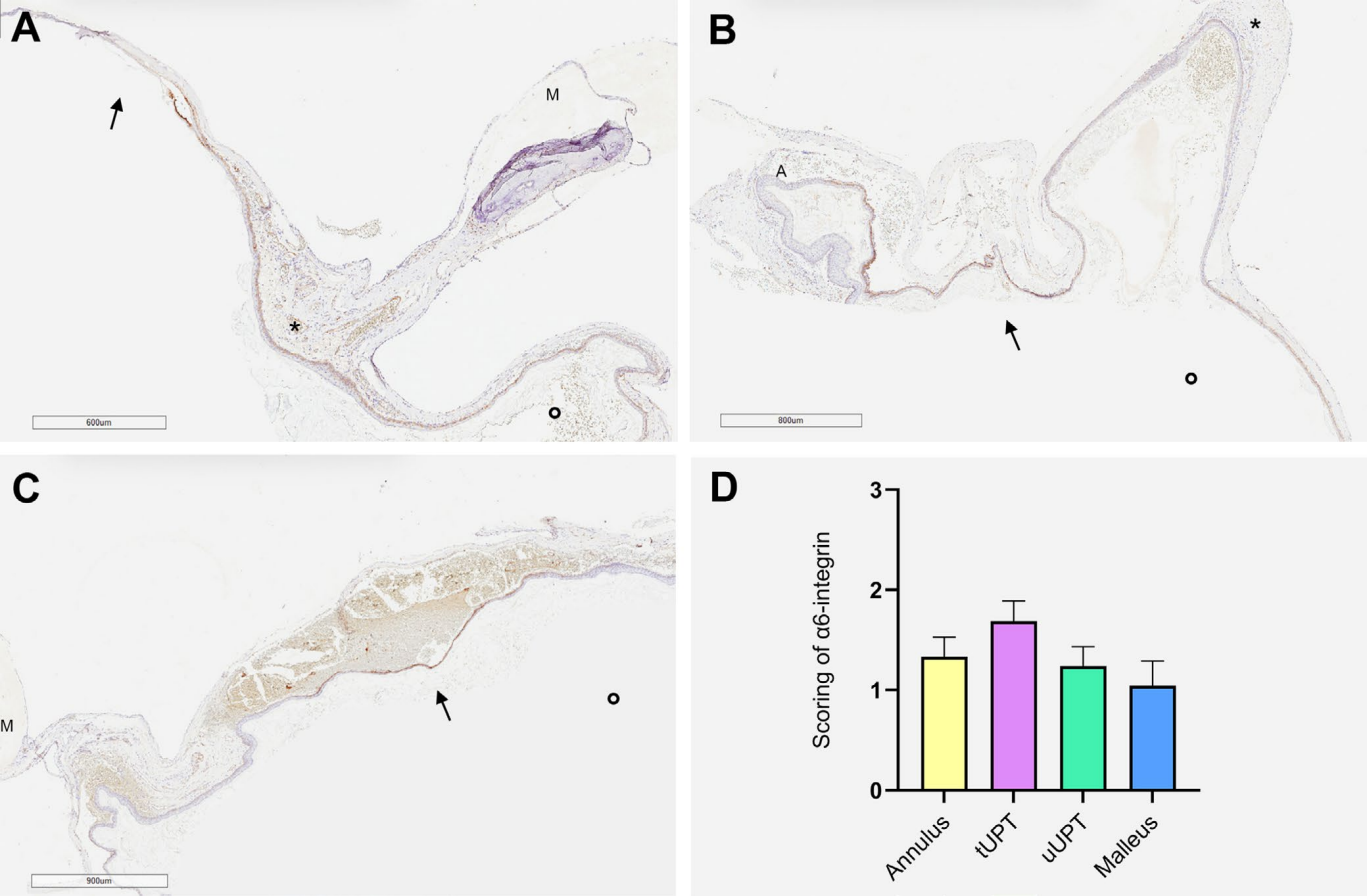
α6‐integrin in the human tympanic membrane after traumatization. (A‐C) Immunohistochemical staining of α6‐integrin in the tympanic membrane 20 h after traumatization. Open circle = external auditory canal, M = handle of malleus, A = annulus, star = region of malleus, arrow = site of traumatization. (D) Evaluation of α6‐integrin staining of tympanic membranes. Each value within each bar (*n* = 16–31) represents one slide with mean scoring, with standard error of mean (*p < 0.05). UPT = unattached pars tensa, tUPT = traumatized UPT, uUPT = untraumatized UPT. [Color figure can be viewed in the online issue, which is available at www.laryngoscope.com]

No overall difference was seen in antibody appearance for the protein markers CK19, Ki‐67, and p63 between the TMs depending on the method of trauma used (Figures S1–S3). When analyzing the TMs separately depending on the method of trauma, the staining intensity of α6‐integrin was significantly higher scored in the tUPT compared to the malleus in the scratched TMs (*p* = 0.0092) (Figure S4). This was not seen in the TMs traumatized with phenol. See Figure 7 for a summary of the results 20 h after traumatization.

**FIGURE 7:**
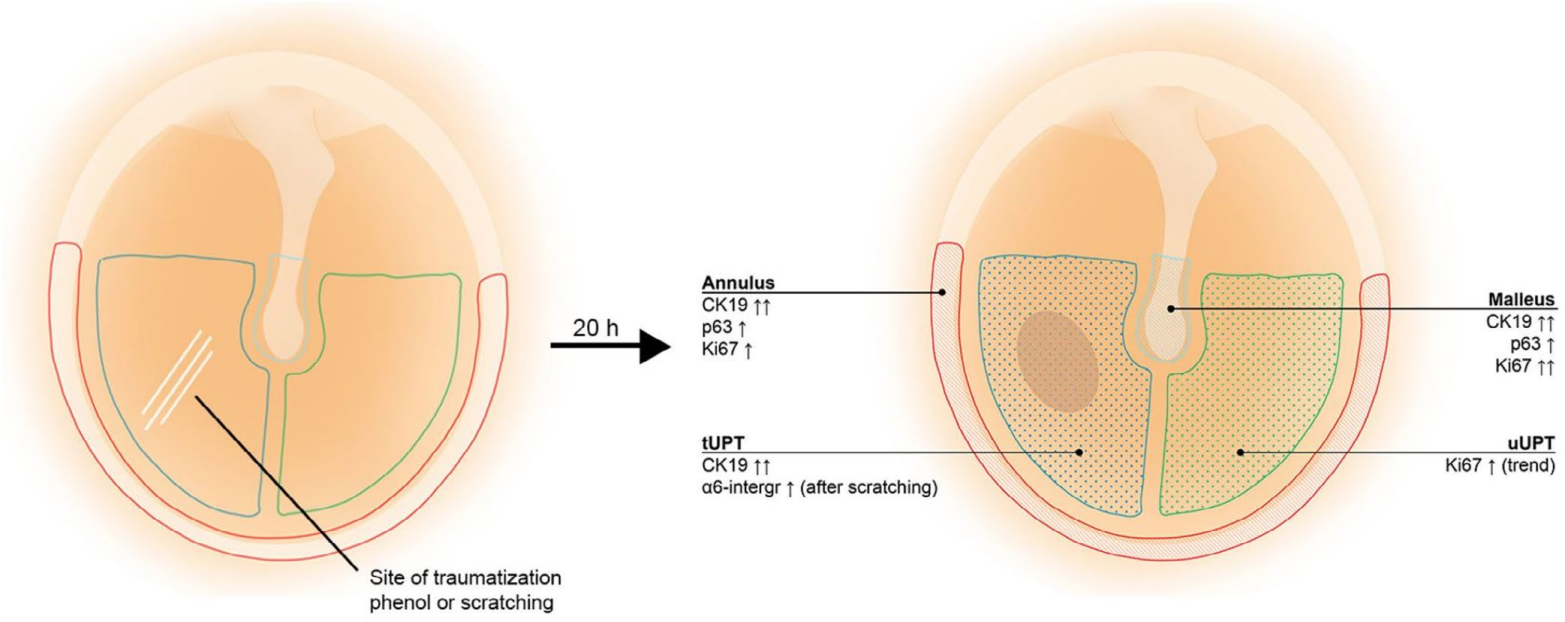
A schematic figure describing the cascade identified in the experimental results 20 h after traumatization. Markers upregulated in one region compared to another highlighted schematically (↑ = trend, *, **; ↑↑ = ***, ****). (*p < 0.05, **p < 0.01, ***p < 0.001, ****p < 0.0001). UPT = unattached pars tensa, tUPT = traumatized UPT, uUPT = untraumatized UPT. [Color figure can be viewed in the online issue, which is available at www.laryngoscope.com]

### 3.3 Regeneration Depending on Anatomical Levels of the Tympanic Membrane

In the uUPT, CK19 was significantly higher scored in the intermediate (the level of the umbo, *p* = 0.0010) and superior (*p* = 0.0071) levels compared to the inferior level (Figure S5). α6‐integrin staining was significantly higher scored in the intermediate (*p* = 0.0255) compared to in the superior level of malleus (Figure S6). There were no significant differences in the scores for the markers between the levels within the tUPT or within the annulus (Figures S7 and S8).

## 4 Discussion

To our knowledge, this is the first study to report on the acute healing response in the keratinocyte layer of human TMs following in vivo traumatization, providing novel insights through the use of an experimental model. Twenty hours after a local trauma, the tUPT showed an increased staining of the epidermal stem cell marker CK19, comparable to the levels of CK19 in the regenerative centers of the annulus and malleus. α6‐integrin, also an epidermal stem cell marker, was upregulated in the scratched cohort in the tUPT. The uUPT, on the other hand, displayed a tendency towards an upregulation in Ki‐67 at the same time point, suggesting a more diverse role of the UPT in the wound healing process than previously suggested [1, 14]. The staining for Ki‐67, p63, and CK19 was generally high in the annulus and malleus regions.

Previous studies with in vivo traumatization on TMs have utilized animal models [1, 2, 8, 14, 15, 16, 17]. One major benefit of an experimental animal setting is the possibility of observational time control. In the present study there was a limitation of the observation time control due to the practical and ethical limitations of the harvest. The aim of the study was to map the early events of a TMP. Hence, the trauma was applied 20 h prior to harvest. To avoid the risk of microbial entrance to the middle ear and thereby an increased risk for wound infection, a decision was made not to perforate the TM completely. This ethical requirement to avoid any increased patient risk related to the research procedure limited the degree of trauma that could be applied and may represent a limitation of our methodological model. To increase the generalizability of the potential findings of the study, two different methods to activate the repair mechanisms of the keratinocytes were used. While phenol is easily applicable and has an anesthetic effect, needle scratching might better mimic a blunt traumatic TMP [18]. Sub‐analyzing according to the traumatization method revealed accepted variabilities between the methods (Figures S1–S4). When stratifying the data by traumatization method, the reduced sample size in each subgroup resulted in a slight overall decline in statistical significance. Despite this, no major differences were observed between the two traumatization methods. Therefore, the results of both methods are relevant and can be suggested for further studies of in vivo traumatization of human TMs.

The TM healing process is divided into three different phases: the inflammatory, the proliferative, and the remodeling phase [1, 6]. The first one, the inflammatory phase, is estimated to last up to 40 h. It starts with opacification and thickening of the TM, both in the connective tissue layer and in the epithelium. This is due to vessel dilatation, reduced cellular adhesion, and inflammatory cell infiltration. The proliferative phase is seen after 24 h with signs of keratinocyte proliferation in the basal layer in the malleus region, but is not seen at or close to the perforation site [1, 2]. The remodeling phase, as seen at the perforation site at 40 h after trauma, exhibits the arrival of migrating keratinocytes, presumably activated during the proliferative phase. In the present study, the healing process was observed at 20 h after traumatization. The main proliferation marker in the present study was Ki‐67. It is widely used clinically for identifying keratinocyte proliferation rate [19]. It was generally highly seen in all regions but significantly most seen in the annulus and malleus regions. These findings verify previously presented data on the malleus and the annulus regions as regenerative centers of the human TM [11]. Twenty hours after the traumatization, Ki‐67 was upregulated in these regions compared to the same regions in the resting TM, suggesting a higher proliferation rate in the traumatized TM in both regions [11]. Simultaneously, the uUPT displayed a tendency towards an upregulation of Ki‐67, suggesting also that the uUPT is involved in the early proliferative event of the repair mechanism.

In skin wound healing, migratory cells appear closest to the site of the wound while proliferation appears further away from the wound site. While epidermal stem cells in skin wound healing are involved in both proliferation and migration, previous studies indicated that migration seems to have a more crucial role in re‐epithelialization than proliferation [20, 21]. This could explain why the normal healing process in the TM, due to its natural migratory capacity, works exceptionally well even with an absence of a natural scaffold. CK19 is a cytoskeletal protein, mainly allocated in the basal layer of the skin, and is considered a specific epidermal stem cell marker and is associated with proliferation [22]. It is also used as a prognostic marker in several epidermal cancer types, partially due to its migrating and metastatic potential [23]. In the present study there was a significant difference in CK19 levels between the uUPT and the tUPT, with higher levels in the pars tensa close to the site of the wound (tUPT). Here, the staining score reached those of the malleus region. This regenerative capacity of the tUPT in this early phase has to our knowledge previously not been described [1, 14]. Its possible importance must be evaluated in further studies. Comparing these results with the CK19 expression in the resting human TM, there is an evident upregulation close to the wound site at 20 h post trauma [11]. Similar upregulation of CK19 was also found in the annulus of the traumatized TMs, compared to in the resting TM [11]. This indicates a possible centripetal migration direction, in contrast to the normal centrifugal migration process in the untraumatized TM.

Integrins are well‐known mediators of cell‐adhesion, migration and epidermal differentiation and play an important role in wound healing in skin [24, 25, 26]. α6‐integrin was detected in all regions of the TM at 20 h after traumatization. There were however significant differences between the regions. When compiling the TMs traumatized by scratching, a significant upregulation of α6‐integrin was seen in the tUPT. There was a tendency towards the same upregulation when analyzing all slides; however, not reaching statistical significance. In the resting human TM, α6‐integrin is significantly more prevalent in the regions of annulus [11]. In the present study, this finding is not evident at 20 h after trauma, but to the contrary, it seems that α6‐integrin is upregulated in the tUPT. Whether this is an effect of α6‐integrin positive cell migration towards the perforation, or of an activation of epidermal progenitor cells in the pars tensa close to the perforation site, is still unclear.

The transcription factor p63 is a nucleoprotein and member of the p53 family. As an epidermal stem cell marker, it is known for its regulating role in epidermal stem cell differentiation and proliferation [27, 28, 29]. A previous study by our group showed the presence of p63 along the entire pars tensa in the human TM, with a higher presence in the annulus and malleus regions [11]. The present study verifies these previous data on regenerative centers of the human TM, with a similar staining pattern regarding anti‐p63 antibody. At the same time, the present findings showed higher levels of p63 in the tUPT and uUPT compared to the levels in the resting UPT [11]. p63 might thus have a role in the early healing events of a TMP through actions in the UPT. Previous publications reveal distinct keratinocyte hierarchies and migratory programs diverse in different levels of the TM [30]. The present study suggests to a limited extent a vertical hierarchy of CK19 and α6 in the uUPT and along the malleus 20 h after traumatization (Figures S5–S8).

To our knowledge, this is the first human study mapping the early repairing events after TM traumatization in vivo. While the use of immunohistochemistry as the sole method represents a limitation, the findings provide a foundation for future studies employing complementary approaches. The results display a snapshot of this dynamic process, advocating future studies focusing on the following events of this healing process. The study is explorative, generating results of basic research confirming findings made in rodents. The results, however, aim to extend to have a clinical implication. Tympanic membrane perforations occur regularly and most often heal spontaneously. Some perforations, however, fail to heal. If the natural healing process could be reactivated in the patients suffering from these remaining cTMPs, their symptoms might be resolved without surgery.

## 5 Conclusion

The present study shows that a trauma to the human TM activates a regenerative process, which involves the activation of proliferative centers similar to the ones shown in the resting TM, the annulus and the malleus regions. The study also reveals a role for the UPT, both close to and further away from the trauma, in the early healing process. The results indicate an upregulated proliferation rate already at 20 h after the trauma, and that the pars tensa also plays a role through activation of keratinocyte progenitor cells. These results should be considered in future studies aiming to improve the TM healing capability, through restoring the normal internal factors in the healing process.

## Funding

Project grants from ALF (Avtal om Läkarutbildning och Forskning) Medicine (J.A.: #FoUI‐1000976), Petrus and Augusta Hedlund Foundation (J.A.: #M‐2024‐2296), the Swedish Society of Medicine (J.A.: #SLS‐998787), the Swedish Association for Otorhinolaryngology Head and Neck Surgery, Syskonen Elsy, Harry and Henrik Johanssons Foundation and the Acta Oto‐Laryngologica Foundation. The funders had no role in the design, data collection, data analysis, and reporting of this study.

## Conflicts of Interest

The authors declare no conflicts of interest.

## Supporting information


**Figure S1:** CK19 in the human tympanic membrane after traumatization (A) through scratching (*n* = 8–11) and (B) through phenol (*n* = 7–24). Each value within the bars (*n*) represents one slide with mean scoring, with standard error of mean (*p < 0.05, **p < 0.01, ***p < 0.001, ****p < 0.0001). UPT = unattached pars tensa, tUPT = traumatized UPT, uUPT = untraumatized UPT.
**Figure S2:** Ki‐67 in the human tympanic membrane after traumatization (A) through scratching (*n* = 9–16) and (B) through phenol (*n* = 7–15). Each value within the bars (*n*) represents one slide with mean scoring, with standard error of mean (*p < 0.05, **p < 0.01, ***p < 0.001, ****p < 0.0001). UPT = unattached pars tensa, tUPT = traumatized UPT, uUPT = untraumatized UPT.
**Figure S3:** p63 in the human tympanic membrane after traumatization (A) through scratching (*n* = 12–18) and (B) through phenol (*n* = 5–17). Each value within the bars (*n*) represents one slide with mean scoring, with standard error of mean (*p < 0.05, **p < 0.01, ***p < 0.001, ****p < 0.0001). UPT = unattached pars tensa, tUPT = traumatized UPT, uUPT = untraumatized UPT.
**Figure S4:** α6‐integrin in the human tympanic membrane after traumatization (A) through scratching (*n* = 10–18) and (B) through phenol (*n* = 6–13). Each value within the bars (*n*) represents one slide with mean scoring, with standard error of mean (*p < 0.05, **p < 0.01, ***p < 0.001, ****p < 0.0001). UPT = unattached pars tensa, tUPT = traumatized UPT, uUPT = untraumatized UPT.
**Figure S5:** Presence of stem cell and proliferative protein markers in different levels of the untraumatized unattached pars tensa (uUPT) region. Evaluation of antibody staining of (A) Ki‐67 (*n* = 7–12), (B) p63 (*n* = 10–13), (C) CK19 (*n* = 10–13), and (D) α6‐integrin (*n* = 7–13) in different levels of the uUPT region in human tympanic membranes, evaluated with immunohistochemistry. Each value within the bars (*n*) represents one slide with mean scoring, with standard error of mean (*p < 0.05, **p < 0.01, ***p < 0.001, ****p < 0.0001). Inferior uUPT = uUPT inferior to the level of umbo, intermediate uUPT = uUPT at the same level as the umbo, superior uUPT = uUPT superior to the level of the umbo.
**Figure S6:** Presence of stem cell and proliferative protein markers in different levels of the malleus region. Evaluation of antibody staining of (A) Ki‐67 (*n* = 9–11), (B) p63 (*n* = 11–13), (C) CK19 (*n* = 10–11), and (D) α6‐integrin (*n* = 9–13) in different levels of the malleus region in human tympanic membranes, evaluated with immunohistochemistry. Each value within the bars (*n*) represents one slide with mean scoring, with standard error of mean (*p < 0.05, **p < 0.01, ***p < 0.001, ****p < 0.0001). Intermediate malleus = malleus at the level as the umbo, superior malleus = malleus superior to the level of the umbo.
**Figure S7:** Presence of stem cell and proliferative protein markers in different levels of the annulus region. Evaluation of antibody staining of (A) Ki‐67 (*n* = 9–12), (B) p63 (*n* = 3–12), (C) CK19 (*n* = 8–13), and (D) α6‐integrin (*n* = 6–10) in different levels of the annulus region in human tympanic membranes, evaluated with immunohistochemistry. Each value within the bars (*n*) represents one slide with mean scoring, with standard error of mean (*p < 0.05, **p < 0.01, ***p < 0.001, ****p < 0.0001). Inferior annulus = annulus inferior to the level of umbo, intermediate annulus = annulus at the same level as the umbo, superior annulus = annulus superior to the level of the umbo.
**Figure S8:** Presence of stem cell and proliferative protein markers in different levels of the traumatized unattached pars tensa (tUPT) region. Evaluation of antibody staining of (A) Ki‐67 (*n* = 3–7), (B) p63 (*n* = 3–7), (C) CK19 (*n* = 2–7), and (D) α6‐integrin (*n* = 2–8) in different levels of the tUPT region in human tympanic membranes, evaluated with immunohistochemistry. Each value within the bars (*n*) represents one slide with mean scoring, with standard error of mean (*p < 0.05, **p < 0.01, ***p < 0.001, ****p < 0.0001). Inferior tUPT = tUPT inferior to the level of umbo, intermediate tUPT = tUPT at the same level as the umbo, superior tUPT = tUPT superior to the level of the umbo.
**Table S1:** Antibody details.

**Table S2:** Calculations of weighted Cohen's kappa coefficient.

## Data Availability

The data that support the findings of this study are available on request from the corresponding author. The data are not publicly available due to privacy or ethical restrictions.
