# Supplementary material for: The Early Regenerative Events in the Human Tympanic Membrane After In Vivo Traumatization
Source: Laryngoscope. 2026 Jul 21;136(12):5511–20. doi: 10.1002/lary.70717 (PMC13765427; doi:10.1002/lary.70717)
Supplement: Supplementary file 1 [file LARY-136-5511-s001.docx]

**SUPPLEMENTARY MATERIAL**

**SUPPLEMENTARY TABLE 1** Antibody details.

| **Primary antibody** | **Product number** | **Company** | **Dilution** | **Antigen retrieval solution** |
| --- | --- | --- | --- | --- |
| Anti-Ki-67 | IR626 | Agilent | Ready-to-use | Low pH |
| Anti-p63 | IR662 | Agilent | Ready-to-use | High pH |
| Anti-CK19 | sc-6278 | Santa-Cruz | 1:50 | High pH |
| Anti-α6-integrin | sc-374057 | Santa-Cruz | 1:25 | High pH |

**SUPPLEMENTARY FIGURE 1** CK19 in the human tympanic membrane after traumatization (A) through scratching (*n* = 8-11) and (B) through phenol (*n* = 7-24). Each value within the bars (*n*) represents one slide with mean scoring, with standard error of mean (**p* < 0.05, ***p* < 0.01, ****p* < 0.001, *****p* < 0.0001). UPT = unattached pars tensa, tUPT = traumatized UPT, uUPT= untraumatized UPT.

**SUPPLEMENTARY FIGURE 2** Ki-67 in the human tympanic membrane after traumatization (A) through scratching (*n* = 9-16) and (B) through phenol (*n* = 7-15). Each value within the bars (*n*) represents one slide with mean scoring, with standard error of mean (**p* < 0.05, ***p* < 0.01, ****p* < 0.001, *****p* < 0.0001). UPT = unattached pars tensa, tUPT = traumatized UPT, uUPT= untraumatized UPT.

**SUPPLEMENTARY FIGURE 3** p63 in the human tympanic membrane after traumatization (A) through scratching (*n* = 12-18) and (B) through phenol (*n* = 5-17). Each value within the bars (*n*) represents one slide with mean scoring, with standard error of mean (**p* < 0.05, ***p* < 0.01, ****p* < 0.001, *****p* < 0.0001). UPT = unattached pars tensa, tUPT = traumatized UPT, uUPT= untraumatized UPT.

**SUPPLEMENTARY FIGURE 4** α6-integrin in the human tympanic membrane after traumatization (A) through scratching (*n* = 10-18) and (B) through phenol (*n* = 6-13). Each value within the bars (*n*) represents one slide with mean scoring, with standard error of mean (**p* < 0.05, ***p* < 0.01, ****p* < 0.001, *****p* < 0.0001). UPT = unattached pars tensa, tUPT = traumatized UPT, uUPT= untraumatized UPT.

**SUPPLEMENTARY FIGURE 5** Presence of stem cell and proliferative protein markers in different levels of the untraumatized unattached pars tensa (uUPT) region. Evaluation of antibody staining of (A) Ki-67 (*n* = 7-12), (B) p63 (*n* = 10-13), (C) CK19 (*n* = 10-13), and (D) α6-integrin (*n* = 7-13) in different levels of the uUPT region in human tympanic membranes, evaluated with immunohistochemistry. Each value within the bars (*n*) represents one slide with mean scoring, with standard error of mean (**p* < 0.05, ***p* < 0.01, ****p* < 0.001, *****p* < 0.0001). Inferior uUPT = uUPT inferior to the level of umbo, intermediate uUPT = uUPT at the same level as the umbo, superior uUPT = uUPT superior to the level of the umbo.

**SUPPLEMENTARY FIGURE 6** Presence of stem cell and proliferative protein markers in different levels of the malleus region. Evaluation of antibody staining of (A) Ki-67 (*n* = 9-11), (B) p63 (*n* = 11-13), (C) CK19 (*n* = 10-11), and (D) α6-integrin (*n* = 9-13) in different levels of the malleus region in human tympanic membranes, evaluated with immunohistochemistry. Each value within the bars (*n*) represents one slide with mean scoring, with standard error of mean (**p* < 0.05, ***p* < 0.01, ****p* < 0.001, *****p* < 0.0001). Intermediate malleus = malleus at the level as the umbo, superior malleus = malleus superior to the level of the umbo.

**SUPPLEMENTARY FIGURE 7** Presence of stem cell and proliferative protein markers in different levels of the annulus region. Evaluation of antibody staining of (A) Ki-67 (*n* = 9-12), (B) p63 (*n* = 3-12), (C) CK19 (*n* = 8-13), and (D) α6-integrin (*n* = 6-10) in different levels of the annulus region in human tympanic membranes, evaluated with immunohistochemistry. Each value within the bars (*n*) represents one slide with mean scoring, with standard error of mean (**p* < 0.05, ***p* < 0.01, ****p* < 0.001, *****p* < 0.0001). Inferior annulus = annulus inferior to the level of umbo, intermediate annulus = annulus at the same level as the umbo, superior annulus = annulus superior to the level of the umbo.

**SUPPLEMENTARY FIGURE 8** Presence of stem cell and proliferative protein markers in different levels of the traumatized unattached pars tensa (tUPT) region. Evaluation of antibody staining of (A) Ki-67 (*n* = 3-7), (B) p63 (*n* = 3-7), (C) CK19 (*n* = 2-7), and (D) α6-integrin (*n* = 2-8) in different levels of the tUPT region in human tympanic membranes, evaluated with immunohistochemistry. Each value within the bars (*n*) represents one slide with mean scoring, with standard error of mean (**p* < 0.05, ***p* < 0.01, ****p* < 0.001, *****p* < 0.0001). Inferior tUPT = tUPT inferior to the level of umbo, intermediate tUPT = tUPT at the same level as the umbo, superior tUPT = tUPT superior to the level of the umbo.
